# Implication of O_2_ dynamics for both N_2_O and CH_4_ emissions from soil during biological soil disinfestation

**DOI:** 10.1038/s41598-021-86026-3

**Published:** 2021-03-23

**Authors:** Chen Wang, Xuehong Ma, Gang Wang, Guitong Li, Kun Zhu

**Affiliations:** 1 Department of Soil and Water Sciences, China Agricultural University, Haidian District, Yuanmingyuan West Road 2, Beijing, 100193 China; 2 Beijing Aogenike Biological Technology Co., LTD, Lisui County, Shunyi District, Gexinlu 3, Gedaizi village, Beijing, 101300 China

**Keywords:** Environmental sciences, Environmental chemistry, Environmental impact

## Abstract

Soil O_2_ dynamics have significant influences on greenhouse gas emissions during soil management practice. In this study, we deployed O_2_-specific planar optodes to visualize spatiotemporal distribution of O_2_ in soils treated with biological soil disinfestation (BSD). This study aimed to reveal the role of anoxia development on emissions of N_2_O and CH_4_ from soil amended with crop residues during BSD period. The incorporation of crop residues includes wheat straw only, wheat straw with biochar and early straw incorporation. The anoxia in soil developed very fast within 3 days, while the O_2_ in headspace decreased much slower and it became anaerobic after 5 days, which was significantly affected by straw and biochar additions. The N_2_O emissions were positively correlated with soil hypoxic fraction. The CH_4_ emissions were not significant until the anoxia dominated in both soil and headspace. The co-application of biochar with straw delayed the anoxia development and extended the hypoxic area in soil, resulting in lower emissions of N_2_O and CH_4_. Those results highlight that the soil O_2_ dynamic was the key variable triggering the N_2_O and CH_4_ productions. Therefore, detailed information of soil O_2_ availability could be highly beneficial for optimizing the strategies of organic amendments incorporation in the BSD technique.

## Introduction

The emissions of greenhouse gases (methane and nitrous oxide) from soils have become a global concern, because methane (CH_4_) is the largest non-carbon dioxide (CO_2_) climate forcing agent^1^ and nitrous oxide (N_2_O) is the most significant ozone-depleting gas in the atmosphere^2^. Agricultural soils contributed to over 47% and 65% of anthropogenic CH_4_ and N_2_O emissions respectively^3,4^. Although the main production processes of CH_4_ and N_2_O in soils are known to be the microbial driven methanogenesis and nitrification/denitrification^5,6^, it still remain challenging to pinpoint the key biological mechanisms and the interactions among environmental variables on CH_4_ and N_2_O production.

One of the crucial factors determining GHG emission is the availability of soil O_2_, which regulated the production and consumption of CH_4_ and N_2_O by influencing the soil redox conditions. Soil O_2_ availability is the net result of the O_2_ consuming and diffusing processes, which is controlled primarily by the microbial substrates and gas diffusivities, such as the bioavailable carbon (C) and nitrogen (N) together with soil moisture and pore structures. Despite the crucial role of O_2_ in regulating the processes of CH_4_ and N_2_O production, very few studies have quantitively investigated the effects of O_2_ on CH_4_ and N_2_O emissions in soils and how such effects are affected by the complex interactions between soil and management factors^7^. Particularly, soil management practices could easily alter soil O_2_ availability^8,9^. For instance, biological soil disinfestation (BSD), which is a common practice for controlling soil-borne diseases in a variety of crops^10,11^, would facilitate fast consumption of O_2_, due to the involvement of the incorporating organic C enriched sources (such as crop residues) into the soil. Furthermore, it greatly reduces the O_2_ diffusion, as it tarps the soil with a plastic mulching film, then irrigating soil to saturation. The O_2_ depletion in soil under the BSD treatment would be mainly determined by the degradability of added organic amendments^12^. The soil O_2_ could additionally come from the diffusion of the O_2_ in the narrow headspace between soil surface and mulching film, which would be depleted as well. The induced O_2_ depletion and/or the accumulation of organic acids produced from the C source decomposition during BSD treatment are suppressive or toxic for several soil-borne pests and plant pathogens^13,14^. The soil environment, temporally shifting from aerobic to anaerobic conditions, was a key factor shaping microbial activities^14,15^ consequently influencing the formation of the C and N gaseous products^16,17^. However, the dynamics of soil O_2_ contents during the entire BSD period have not been investigated in detail at all. Currently, there is a critical gap in our understanding of how BSD-induced soil O_2_ variation impact GHG emissions. Besides, BSD has been adopted as a reliable remediation method but high N_2_O emission would occur in nitrate-riched soils during BSD treatment^18^. The application of biochar could possibly mitigate N_2_O emissions from agricultural soil through the increase of soil pH^19^. However, the biochar’s mitigation effects on N_2_O emission during BSD process has not been fully investigated yet.

In this study we investigated the dynamics of the soil O_2_, the emissions of CH_4_ and N_2_O during the entire BSD treatment period. The specific objectives were: (1) to investigate the effects of the crop residue incorporation on soil O_2_ dynamics during BSD treatment period; (2) to link the BSD induced soil O_2_ variation to the emissions of CH_4_ and N_2_O from soil. The different methods of crop residue incorporation included: wheat straw only, wheat straw with biochar, and early straw incorporation.

## Materials and methods

### Experimental sites and treatments

The experiment was conducted in a greenhouse at Shunyi experimental station, China Agricultural University, Beijing, China. Four treatments had been established: soil without any amendments was served as control (CON); soil amended with wheat straw (WH), soil amended with wheat straw 5 days before the BSD treatment (WH + 5D), soil amended with wheat straw and biochar (WH + BC). The four treatments were arranged in a randomized complete block design with 3 replicates.

Straw berry was the dominant crops planted in the greenhouse. Soil was collected from the 0–20 cm layer after harvest of catch crop (maize). The soil was freshly passed through a 2-mm mesh sieve. 8.5 kg of soil (dry matter basis) was repacked into a PVC column (diameter of 20 cm and length of 21 cm) at a bulk density of 1.35 g cm^−3^. A nylon mesh (0.2-mm pore size) was used to cover the bottom of the column, assisting the soil repacking process and column transportations. The application rate of wheat straw and biochar was 10 g kg^−1^ and 5 g kg^−1^ soil respectively, corresponding to rates of 1.62 × 10^4^ kg ha^−1^ and 0.81 × 10^4^ kg ha^−1^ respectively. The added wheat straw was grounded to be less than  2 mm in length. A mixture substrate of rice husks (70%) and cotton seed hulls (30%) was pyrolized at 400 °C for 4 h in a sealed oven to produce the biochar in this experiment. The properties of the soil, straw and biochar were shown in Table S1.

All of the repacked soil columns were placed in a shallow water tank for 6 h, conditioned to reach the moisture content of water-holding capacity. A polyethylene film (0.04-mm thick) was used as the mulch film for soil columns, sealed with silicon glass to make it airtight. There were always some gaps or headspaces between the mulching film and soils because of the uneven soil surface in situ. Therefore, we kept the top 1.0 cm in each column as the headspace with a volume of 0.31 L. Then soil columns were transported to the greenhouse and buried there to keep the soil surface level the same as the ground level.

### Soil sampling

Soil samples were taken at 10 and 30 days with a core sampler (diameter of 2.0-cm). Additional samples were taken at day 0 for treatment WH + 5D. 10 g of each fresh soil sample was used for gravimetric water content determination (drying at 105 °C for 24 h). Another 10 g soil was extracted with 50 mL 1 M potassium chloride (KCl) solution for 1 h and filtered through Whatman no.1 paper. Extracts were frozen at − 20 °C and analyzed later by a Flow Injection Analyser (FIAstar 5000 Analyzer, FOSS, Denmark) for NH_4_^+^ and NO_3_^-^ analysis. The dissolved organic C (DOC) and dissolved organic N (DON) in soil were measured after mixing soil with deionized water (1:5 soil water ratio), shaking for 1 h and then the mixture was filtered through a 0.45 μm microporous film and determined by TOC/TN analyzer (LiquiTOCII, Elementar, Germany).

### Gas sampling

Gas samples were taken from the headspace of each soil column using gas-tight syringes at 0, 0.5, 1, 2, 3, 4, 6, 9, 12, 17, 23 and 30 days for CH_4_, CO_2_ and N_2_O analysis. For each sampling event, 10 mL samples were taken at 0 and 30 min after the start of sampling. All gas samples were analyzed using a gas chromatograph (Clarus 690 GC, PerkinElmer, the United Kingdom) configured with two detectors: an electron capture detector (ECD) and a flame ionization detector (FID). The gas sample was separated by a Porapak QS column (2.0-m). The ECD was set up for N_2_O analysis, the temperature of the oven and detector were 50 and 350 °C respectively, and argon was used as the carrier gas. The FID channel was set up for CH_4_ analysis. CO_2_ was reduced to CH_4_ by a methanization module and then was detected by FID as well. The temperature of the oven and detector were 50 and 370 °C respectively and the carrier gas was helium.

The temperature of soil (0–10 cm) and surface air was measured with a multi-channel soil thermometer (SYS-21G, SAIYASI, China). The relative humidity levels in the air of the greenhouse was monitored with a hydro-thermometer (TES-1360, HUANYU, China).

### Soil O_2_ monitoring

A parallel experiment was conducted in laboratory to assess the dynamics of soil O_2_ using O_2_-specific planar optodes at 36 ℃, which was the average temperature of the soil during BSD treatment. The designed treatments were the same as those in the greenhouse. Soil was incubated in rectangular boxes (height × width × length = 21 cm × 12 cm × 5 cm) suitable for planar optode system. The measuring system of planar optode was adapted from Larsen et al.^20^. Briefly, Pt(II)-tetrakis (pentafluorophenyl) porphyrin (PtTFPP) was used as an O_2_ quenchable luminophore, which was combined with the coumarin dye MACROLEX fluorescence yellow 10GN as an antenna dye. The platinum (II) complex (1% by weight) and the antenna dye (2%) were dissolved in a 10% polystyrene matrix, where toluene was used as the solvent. The mixed solution was then spinning coated onto the 2-mm thick glass inserts (15 × 10 cm), which was fitted into the center of the front window of the acrylic box. A LED assembly was used as the excitation light source. It consisted of seven light-emitting diodes with λ-peak of 447.5 nm (SR-02-R0500, Luxeon Star, Canada). A short-pass filter (475 nm) (Genxu Optics, China) was equipped in front of the excitation light. A long-pass emission filter (Genxu Optics, China) covered the high-quality prime macro lens (SIGMA 50 mm F2.8 EX DG MACRO) to remove any reflected blue light from the excitation source. The O_2_ contents were quantified with the ratio between the red and green luminescence emitted from the indicator and antenna dye respectively. Optode images were taken by a Canon EOS 760D camera, at 30-min intervals for the initial 7 days, and 60-min intervals for the rest of experimental period. The O_2_ levels calculated from the sensing area was used to evaluate the temporal patterns of O_2_ variations in soil and headspace.

### Permeation of CH_4_, CO_2_ and N_2_O through the plastic mulching films

The plastic film used in the BSD was not completely impermeable for CH_4_, CO_2_ and N_2_O. In order to estimate the full fluxes of gases, the permeation of the plastic film for gases was measured by additional laboratory experiments, through a modified method of Nishimura et al.^21^. Briefly, the penetration chamber consists of two 1.0 L cylindrical glass compartments, into which a plastic film is inserted. Round rubber rings on both sides of the film together with silicon glass were used to connect and seal the two compartments tightly. 10 mL of a standard gas including CH_4_, CO_2_ and N_2_O (199 ppmv, 10,100 ppmv and 145 ppmv respectively) was injected into one compartment. After 1, 3, 6 and 12 h, 3.0 mL of the gas from both compartments was drawn respectively with a syringe and injected into the gas chromatograph (Clarus 690 GC, PerkinElmer, the United Kingdom) for gas quantification. Those permeation measurements were carried out in an incubator at 20, 30, 40, 50 and 60 °C under 100% of relative humidity. Three replicates under each temperature were conducted.

### Data analysis

The ratio-metric approach was used for the O_2_ optode measurement^20^. The free software ImageJ (http://rsbweb.nih.gov/ij/) was used to process the recorded optode images. The O_2_ contents in the soil and headspace were calculated from the optode images covering a soil area and headspace area of 10 × 10 cm and 10 × 1 cm respectively, with a unit of % air saturation.

The gas permeation through plastic film was temperature-dependent, which can be considered as an activated energy process and can be well expressed by an Arrhenius-type model, therefore the CH_4_, CO_2_ and N_2_O fluxes to the atmosphere by permeation was then estimated using a modified equation from Stern et al.^22^ :

1 $$F = - D \left( T \right) \frac{\Delta C}{{\Delta x}} = F_{0} \cdot \exp \left( { - \frac{{E_{a} }}{RT}} \right) \cdot \left( {p_{below} - p_{above} } \right)$$

where *F* is the gas (CH_4_, CO_2_ or N_2_O) flux through the film (μg m^−2^ h^−1^), *D* is the diffusive permeability coefficient (m^2^ h^−1^) of the film (temperature-dependent), *ΔC* is the difference of gas concentrations (mg m^−3^) above and below the film, and *Δx* is the thickness of the film (m). *T* is the absolute temperature (K). *E*_*a*_ is the activation energy (J mol^-1^), *R* is the universal gas constant (8.314 J mol^−1^ K^−1^), *T* is the absolute temperature (K), and *F*_*0*_ is an Arrhenius constant (mg m^−2^ h^−1^ atm^−1^). The parameters *E*_*a*_ and *F*_*0*_ were obtained by the above-mentioned permeation experiment. *p*_*below*_ and *p*_*above*_ were the partial pressure of the gas below the mulch film and the ambient gas partial pressure respectively. The ambient gas partial pressures above the film were assumed to be stable (because of the large volume in the greenhouse) during the experimental period, which were fixed to 0.17 Pa, 40.53 Pa, 0.04 Pa for CH_4_, CO_2_ and N_2_O respectively, equivalent to 1700 ppbv, 400 ppmv and 400 ppbv for CH_4_, CO_2_ and N_2_O respectively.

Global warming potential (GWP) was calculated to compare the effects of BSD treatment with different crop residue incorporation. GWP in CO_2_-e per m^2^ was estimated with a GWP of 28 for CH_4_ and 265 for N_2_O^23^:

2 $${\text{GWP}} = 28 \times {\text{R}}\left( {{\text{CH}}_{4} } \right) + {\text{R}}\left( {{\text{CO}}_{2} } \right) + 265 \times {\text{R}}\left( {{\text{N}}_{2} {\text{O}}} \right)$$

where R (CH_4_), R (CO_2_) and R (N_2_O) are the cumulative emissions of CH_4_, CO_2_ and N_2_O (mg m^−2^) during BSD treatment period, respectively.

### Statistic analysis

Differences in average soil O_2_, headspace O_2_, CH_4_, CO_2_ and N_2_O concentrations between different treatments were analyzed by a one-way ANOVA procedure at *p* < 0.05. The Tukey test was used for multiple comparisons. Pearson’s bivariate correlation implied the relationships between different measured variables.

### Ethics approval and consent to participate

Not applicable.

### Consent for publication

Not applicable.

## Results

### Climate conditions of soil and air in the greenhouse

**Figure 1:**
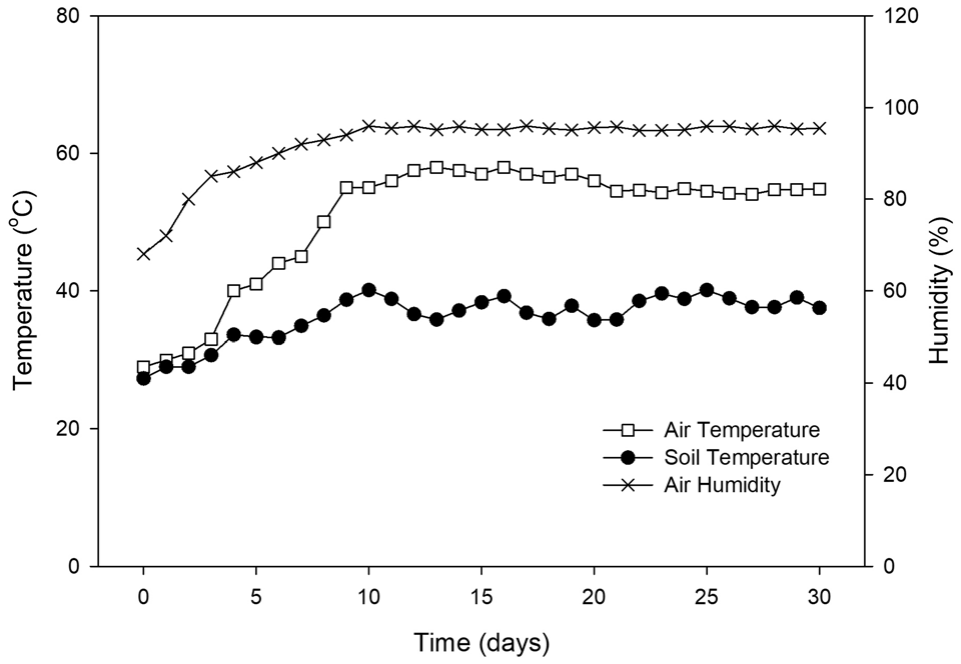
The temperature (soil and air) and relative humidity (air) in the greenhouse during the BSD treatment.

During the initial 7 days, both temperatures and relative humidity of the air in the greenhouse sharply increased (Fig. 1). The peak temperature was reached at day 12 with 58.0 ℃. It was kept in a quite stable condition with 2 degrees of fluctuation. After 10 days, the relative humidity was the highest with 96% until the end of the experimental period. The soil temperature gradually increased from 26 to 40 ℃ within 10 days, and then was maintained at 36 ± 3℃.

### Available N and C in soil

**Figure 2:**
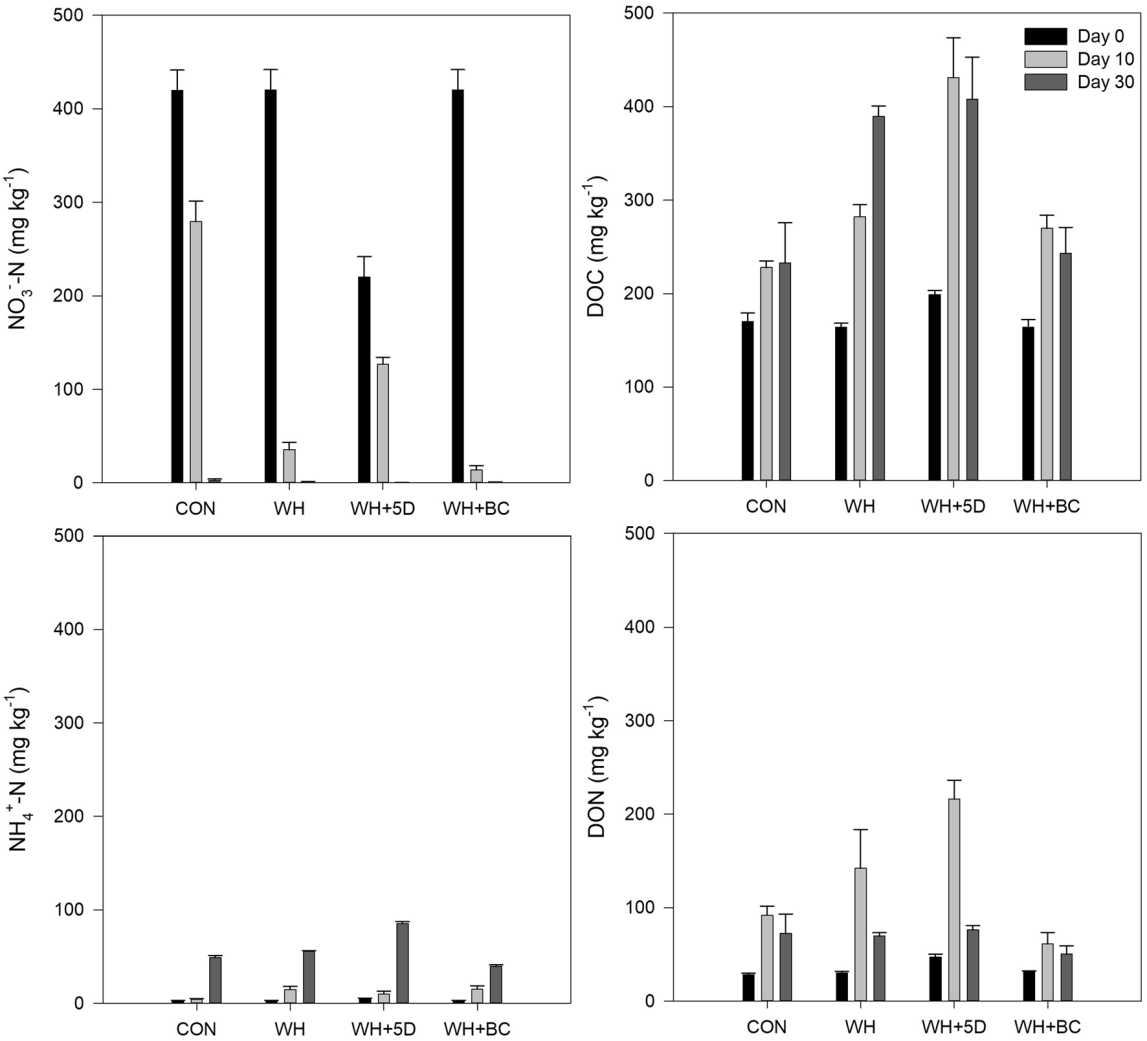
Nitrate, ammonium, DOC and DON contents in soil during the BSD treatment period. CON: soil without any amendments; WH: soil amended with wheat straw; WH + 5D: soil amended with wheat straw 5 days before the BSD treatment; WH + BC: soil amended with wheat straw and biochar.

The BSD treatment had significant effects on N and C availabilities in soil (Fig. 2). Nitrate was consumed dramatically and depleted completely at the end of the BSD treatment. The addition of wheat straw accelerated this depletion process. Particularly, the early application of wheat straw consumed half of the native soil nitrate during the 5 days of aerobic treatment. Biochar addition coupled with wheat straw had the highest nitrate depletion rate during the first 10 days of BSD treatment.

Contrast to nitrate dynamics, soil ammonium increased gradually, and the treatment of WH + 5D resulted in the highest ammonium content. The addition of wheat straw increased the ammonium accumulation rate during the first 10 days period, which, however, was not significant at the end of BSD treatment. Similarly, the WH + BC followed the same trend as the wheat straw-only addition in terms of ammonium nitrogen changes over the first ten days, but at the end WH + BC had the lowest ammonium nitrogen content among the four treatments. The DOC dynamics was quite similar to the ammonium, the only difference was that DOC accumulation mainly occurred in the first 10 days of BSD treatment.

Soil DON contents increased during the initial 10 days but declined at the end. Biochar addition coupled with wheat straw resulted in the lowest DON production.

### Oxygen dynamics in soil and headspace

**Figure 3:**
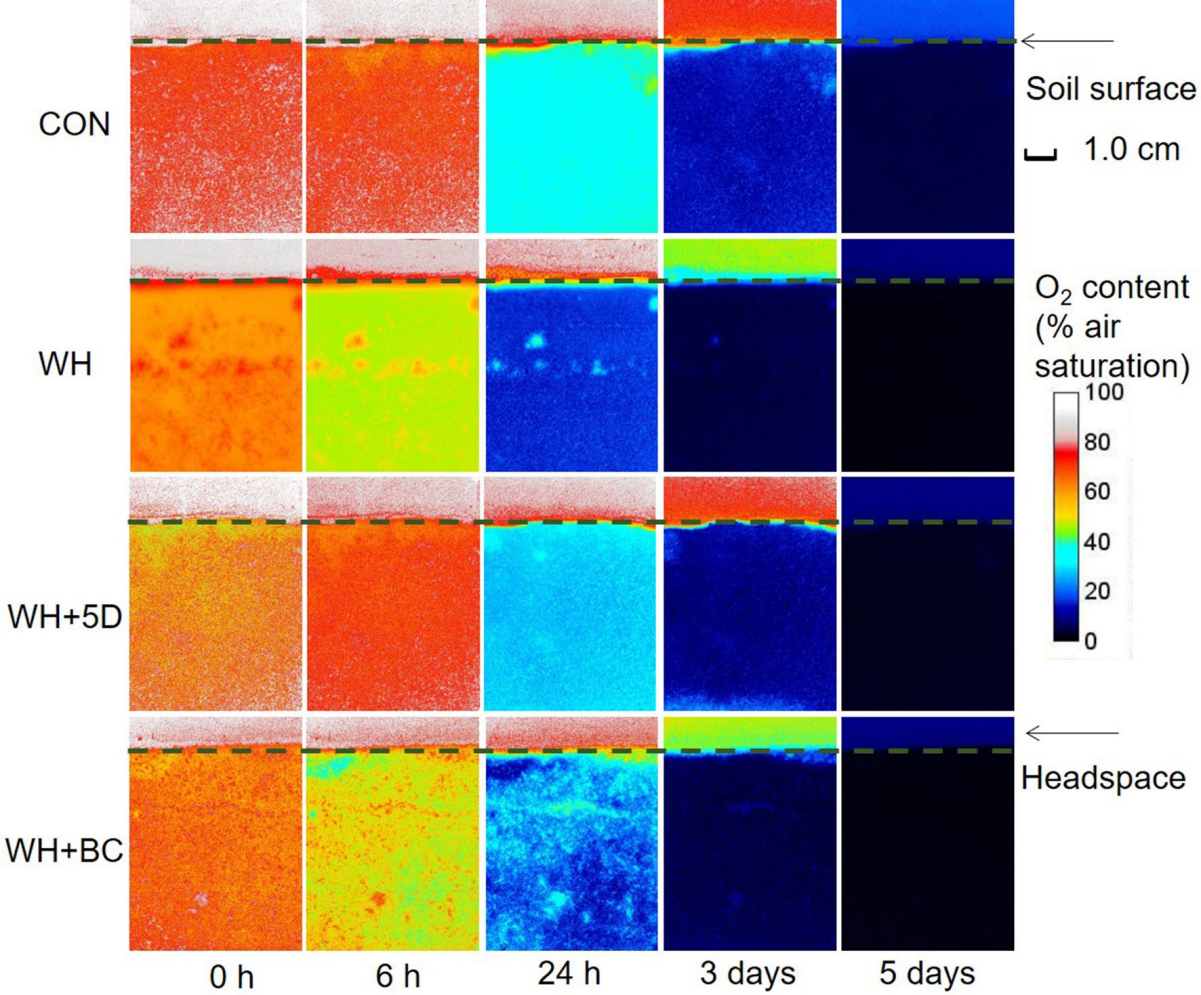
Selected images of O_2_ content in soil and headspace after different straw applications. Images are one representative example of the three replicates. CON: soil without any amendments; WH: soil amended with wheat straw; WH + 5D: soil amended with wheat straw 5 days before the BSD treatment; WH + BC: soil amended with wheat straw and biochar.

Generally, the O_2_ contents dropped dramatically within 5 days in all treatments. Initially, the soil O_2_ contents were 94% air saturation in CON (Fig. 3), slightly higher in straw-amended treatments (around 90% air saturation). This indicated that a small proportion of soil O_2_ had already been consumed in straw-amended treatments during the setting up period (about 60 min). After 3 days, the mean O_2_ contents in soil decreased sharply to less than 10% air saturation in all treatments. On average, the O_2_ decreasing rates increased as the following order: CON < WH + 5D < WH + BC < WH, which indicated the significant influences of amendments on soil O_2_ consumption. The O_2_ contents in headspace decreased much slower than that in soil. Until 5 days later, the headspace became anoxic (less than 3% air saturation). The crop residue incorporation had similar effects on the O_2_ contents in headspace and soil: WH treatment resulted in the highest O_2_ consumption rate in headspace.

**Figure 4:**
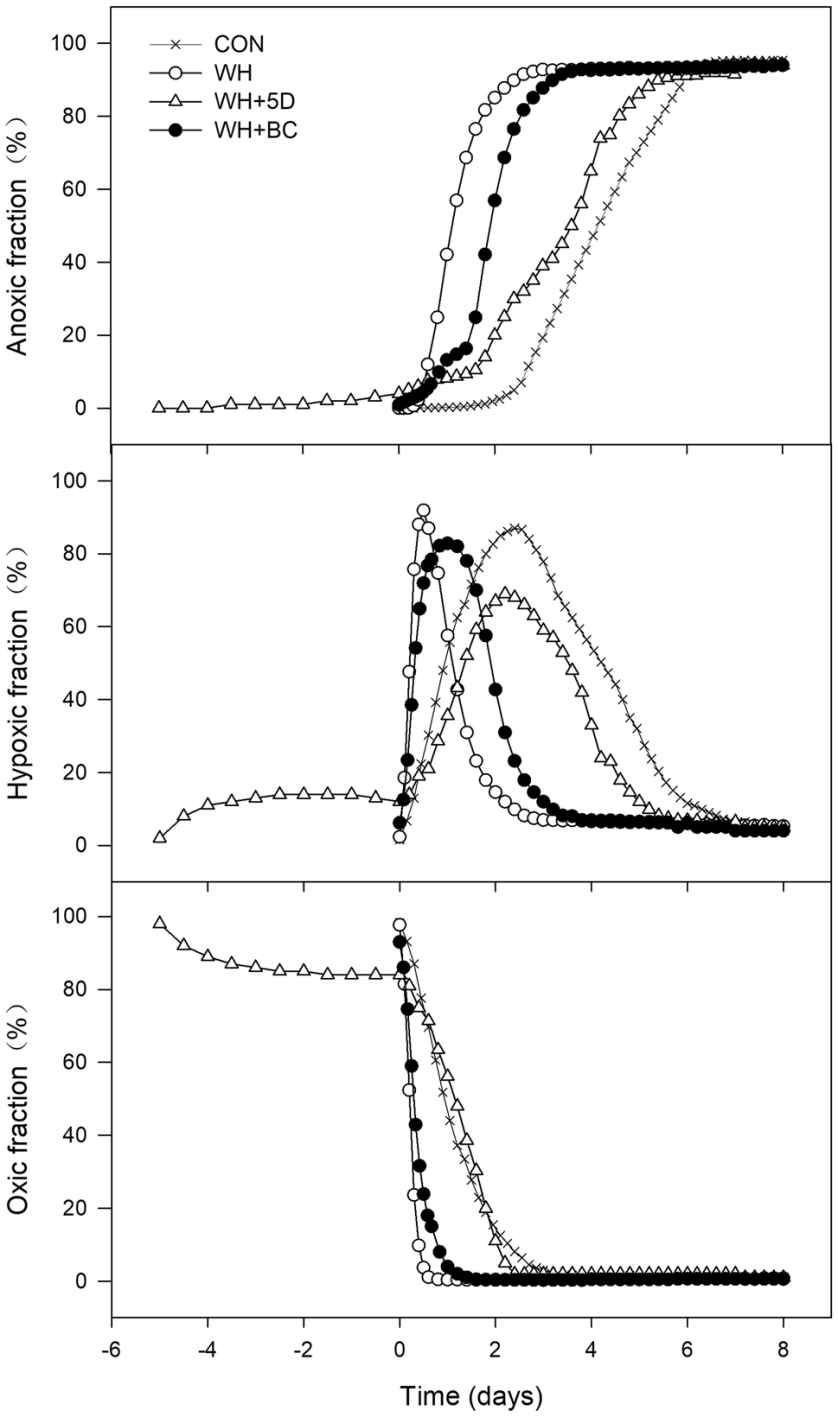
The area fractions of anoxic, hypoxic and oxic conditions (< 0.14, 0.14–2.00, and > 2.00 mg O_2_ L^−1^ respectively) in the soil during the BSD treatment period. CON: soil without any amendments; WH: soil amended with wheat straw; WH + 5D: soil amended with wheat straw 5 days before the BSD treatment; WH + BC: soil amended with wheat straw and biochar.

The levels of soil O_2_ contents could be divided into three categories based on physiological considerations: oxic conditions where the O_2_ content is > 2.00 mg L^−1^, hypoxic with an O_2_ content between 0.14 and 2.00 mg L^−1^, and anoxic conditions with O_2_ content < 0.14 mg L^−1^. Briefly, an O_2_ content below 2.00 mg L^−1^ influences many bio-organisms in terms of behaviour, growth and reproduction^24^; The reduction of elements such as N and Fe, would dominate with anaerobic metabolic process under the conditions of O_2_ content below 0.14 mg L^−1^^25^; with further O_2_ decline (< 0.14 mg L^−1^), methanogenesis may occur as well^26^. Using the obtained optode images, the fractions of anoxic, hypoxic and oxic conditions in all treatments could be calculated. Anoxic area developed the slowest in CON during the entire BSD period (Fig. 4). The WH treatment exhibited the most rapid development of anoxia. The expansion of anoxic area in WH + BC was slightly slower than in WH + 5D initially, however, after 20 h, it became much greater in WH + BC than WH + 5D. The peak fractions of hypoxic area occurred the earliest in WH (0.5 day), followed with WH + BC (1 day) and WH + 5D (2 days). The decline of the oxic fraction in WH was faster than in others and remained lower during the BSD period. Before BSD process, the oxic fraction decreased by 20% in WH + 5D during the initial 5 days of incubation. Once BSD process began, the declining rate of oxic fraction in WH + 5D increased extensively.

### The permeation of CH_4_, CO_2_ and N_2_O fluxes through mulch film

**Figure 5:**
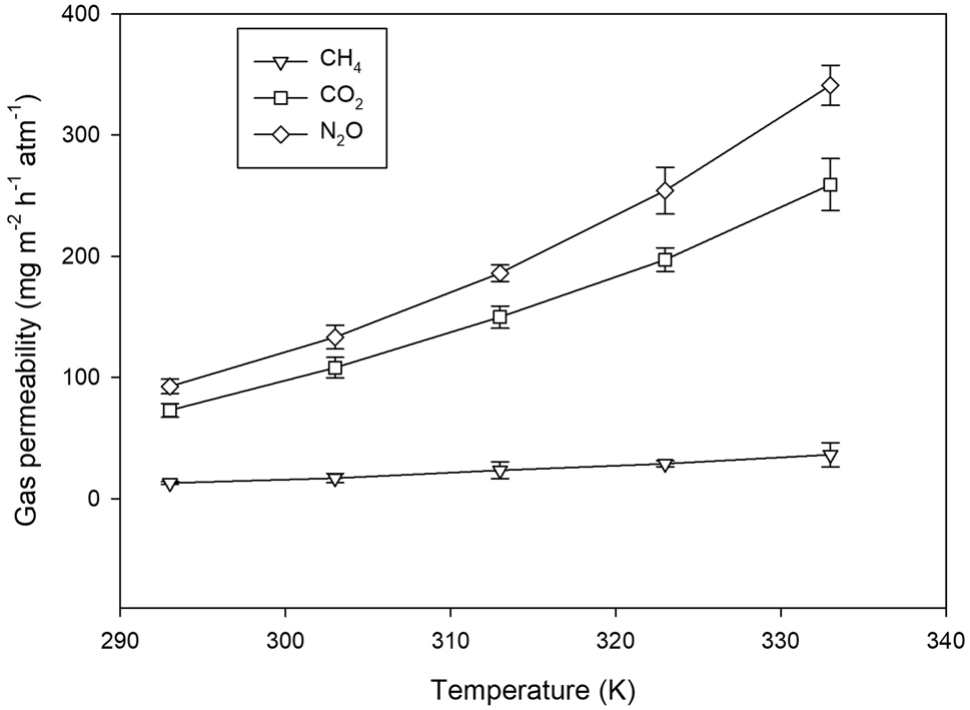
Gas permeability of plastic mulch films as affected by temperature.

As the temperature increased from 20 ℃ to 60 ℃, the gas permeabilities of the mulch film increased exponentially (Fig. 5), which was fitted well with Arrhenius models (Table S2). N_2_O was characterized by the highest gas transmission, which was 9.4 times and 1.3 times as high as that of CH_4_ and CO_2_ respectively. The estimated activation energy was the highest from N_2_O as well. The CH_4_, CO_2_ and N_2_O fluxes by permeation through mulch film were not negligible. In the following sections, the emission rates of CH_4_, CO_2_ and N_2_O from all treatments at every sampling time were corrected with the permeance of respective gases. In addition, by the trapezoidal integration of the estimated flux data during the experiment, we calculated the cumulative emission of each gas by permeation through the mulch film, which accounted for 2.5 – 2.7%, 12.3 – 14.2% and 7.4 – 15.0% of total cumulative emissions of CH_4_, CO_2_ and N_2_O respectively (Fig. 7).

### CH_4_ emissions

**Figure 6:**
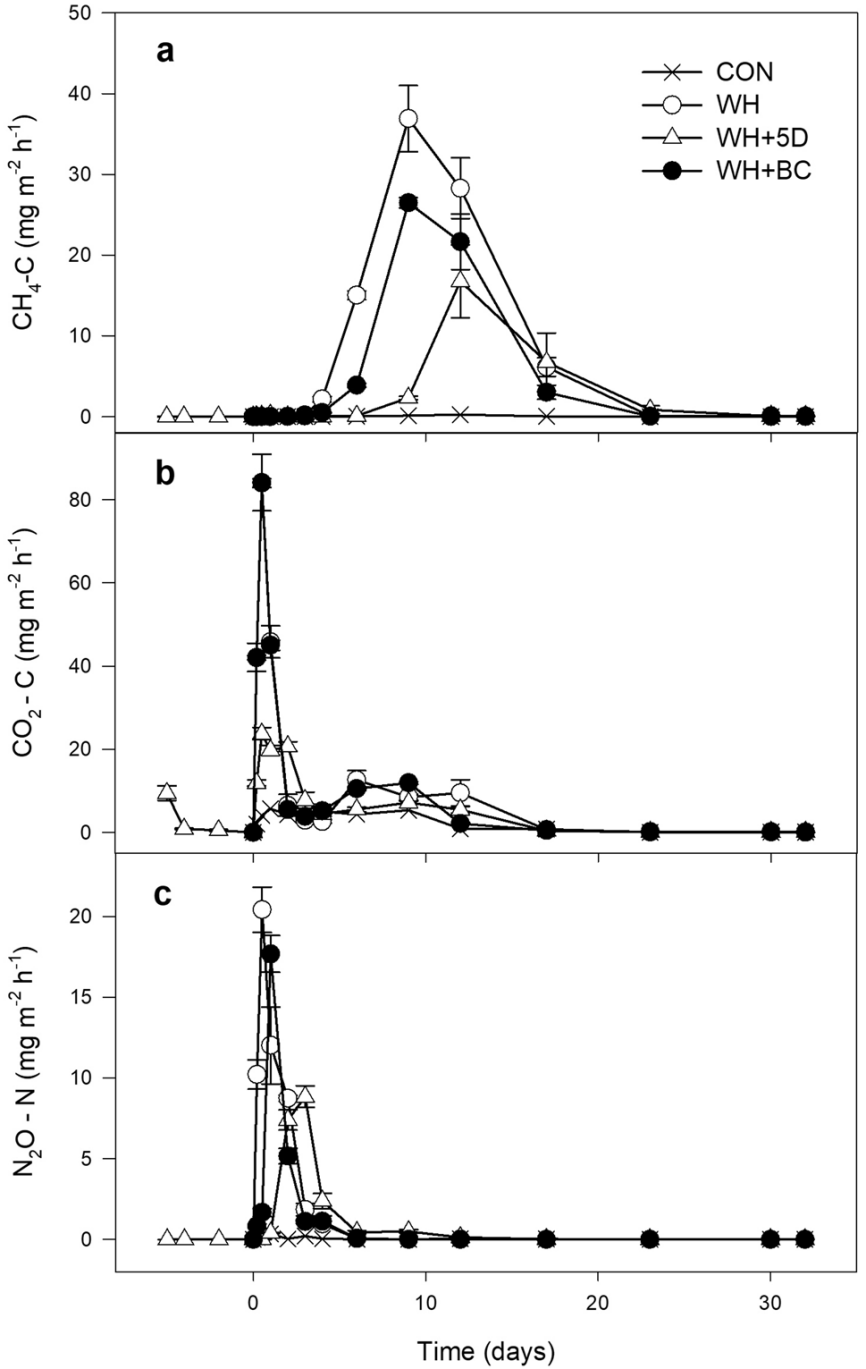
Dynamics of CH_4_, CO_2_ and N_2_O emissions during BSD treatment period. CON: soil without any amendments; WH: soil amended with wheat straw; WH + 5D: soil amended with wheat straw 5 days before the BSD treatment; WH + BC: soil amended with wheat straw and biochar.

**Figure 7:**
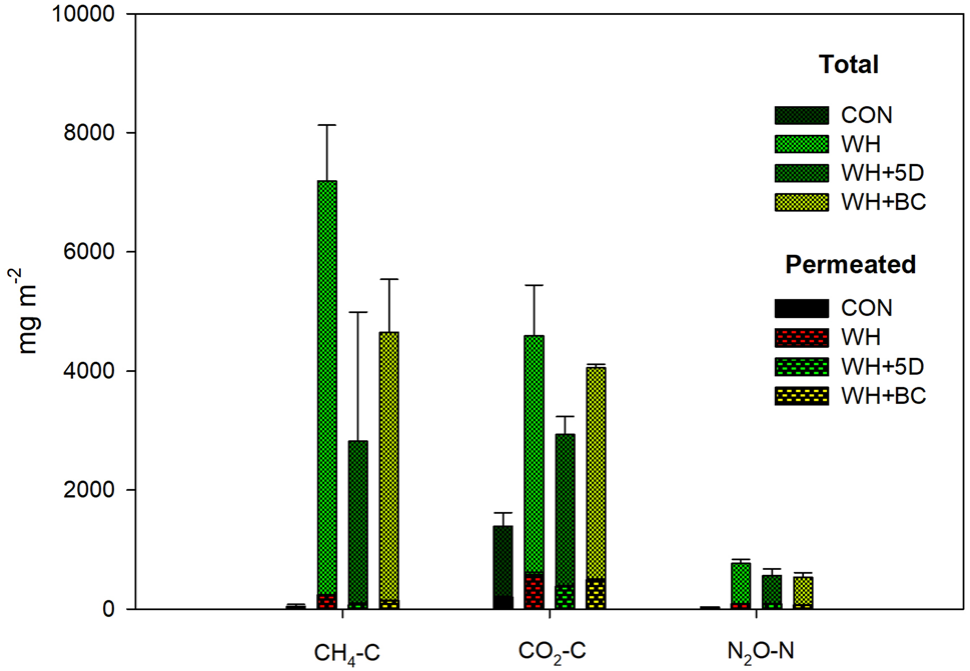
The cumulative emissions (mg m^−2^) of CH_4_, CO_2_ and N_2_O from different amendments during BSD treatment period. Bars with dash-filled pattern were the cumulative emissions of gases permeated through the cover film. CON: soil without any amendments; WH: soil amended with wheat straw; WH + 5D: soil amended with wheat straw 5 days before the BSD treatment; WH + BC: soil amended with wheat straw and biochar.

The temporal patterns of CH_4_ emissions were significantly different among treatments (Fig. 6a). Generally, CH_4_ emissions from all treatments were negligible in the first 3 days, and CON maintained low fluctuating emission rates during the entire experimental period. After 4 days, the emission rates in WH treatment were significantly higher than the other treatments and the highest emission peak was observed after 9 days. The temporal pattern of CH_4_ emission from treatment WH + BC was quite similar as that from WH, but with significantly lower emission rates. The WH + 5D treatment induced the latest emission peak (at day 12) as well as the lowest peak emission rate. The correlation analysis revealed that CH_4_ emission rates was positively correlated with anoxic fractions of soil (Table S3). The cumulative CH_4_ emission during the entire BSD treatment was the largest from the WH treatment, followed by WH + BC and WH + 5D treatment (Fig. 7).

### CO_2_ emissions

The emission rates of CO_2_ increased dramatically at the beginning of the experiment and reached the peaks within 24 h for all treatments (Fig. 6b). The emissions peaked after 12 h in both treatment of WH and WH + BC, followed by a sharp decrease afterwards. The highest emission rate in treatment of WH + 5D was observed after 12 h but followed with significantly slower decreasing rates. Except for day 2 and day 3, CO_2_ emission rates from WH + 5D were consistently lower than those of WH and WH + BC during the BSD treatment period. At day 4, the air temperature in the greenhouse changed sharply from 35 ℃ to 50 ℃, consequently, the CO_2_ emission rates from all treatments increased slightly. As expected, CO_2_ emission rates was observed a negative correlation with soil anoxic fractions, while hypoxic fraction of soil was positively correlated with CO_2_ emission rates (Table S3). The cumulative CO_2_ emission was the highest from the WH treatment and intermediate with the WH + BC treatment, and the lowest from the WH + 5D treatment (Fig. 7).

### N_2_O emissions

N_2_O emissions were influenced by the different amending methods of wheat residue in the soil (Fig. 6c). The N_2_O emission rates increased extensively and reached to the emission peaks within 3 days in straw-amended treatments. The CON had rather low peak emission rate. The earliest and highest emission peak occurred after 12 h from the WH treatment, followed by the WH + BC (peak after 24 h) and WH + 5D treatments (peak after 72 h). The emissions decreased dramatically in treatments of WH and WH + BC after 2 days, while the WH + 5D treatment was observed with relatively longer period of peak emission (about 4 days). Significant correlation (positive) was detected between soil hypoxic fraction and N_2_O emission rates (Table S3). The cumulative N_2_O emissions for the entire BSD treatment period from the WH treatment was the largest, which was 36.5% and 43.2% higher than that from the WH + BC and WH + 5D treatment respectively (Fig. 7).

### Global warming potential

**Figure 8:**
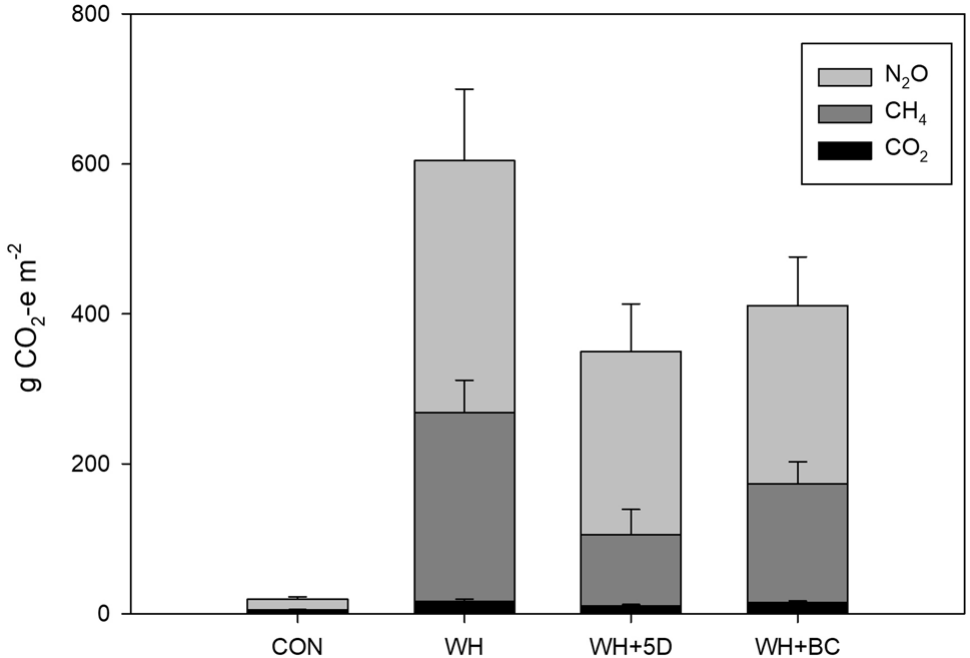
The GWP (g CO_2_-e m^−2^ soil) from different amendments during BSD treatment period. CON: soil without any amendments; WH: soil amended with wheat straw; WH + 5D: soil amended with wheat straw 5 days before the BSD treatment; WH + BC: soil amended with wheat straw and biochar.

The GWP was the largest in treatment of WH, which was 1.81 and 1.50 times as high as that in treatments of WH + 5D and WH + BC, respectively (Fig. 8). The CO_2_ emissions contributed to only 1.8–2.5% of the GWP in all treatments. The contributions of each gas to the GWP varied among treatments. The N_2_O emissions (52.5%) contributed slightly more compared to CH_4_ (45.5%) in treatments of WH, which was the same pattern in treatment of WH + BC. However, the proportion of CH_4_ emissions became much less (29.6%) and N_2_O emissions contributed the main part of GWP (68.6%) in treatment of WH + 5D.

## Discussions

### Effects of crop residue incorporation on O_2_ dynamics during BSD process

The depletion of soil O_2_ contents was significantly influenced by the application of the BSD treatment. The formation of anaerobic conditions during this treatment had regulating impacts on its efficacy^27^. Deploying optode technology enables monitoring the dynamics of anoxia development both in the soil and headspace during BSD process. Addition of organic amendments coupled with sufficient irrigation simultaneously enhanced microbial O_2_ consumption and physical inhibition of O_2_ diffusion^28^. The completely anoxic bulk soil environment developed after 3–5 days. Although the soil was supposed to be tightly covered by the mulching film, there were always some spaces in situ between the film and soil due to the rough soil surface. Such headspace is quite important as a O_2_ source in the initial stage of BSD process. The O_2_ could diffuse from the headspace air into the soil, and the diffusion rates were dependent on the soil gas diffusivity and the gradients of O_2_ contents between headspace and soil. The crop residue addition could possibly improve the gas diffusivity of soil and increase the soil O_2_ consumption as well^29,30^, therefore, it took the longest period (more than 7 days) to reach the anoxic condition in the headspace and soil at the CON treatment. It was worth noting that there were still 1–2% air saturation of O_2_ in headspace and 1–4% of hypoxic area in all soils after the O_2_ status was relatively stable. The O_2_ in the air could possibly permeate through the covered plastic film^31^ and diffuse into the headspace as well as the soils. Such slow O_2_ permeation could be one of the sources for the soil O_2_. In addition, some tortuous pores in soil may trap some gases which were not accessible to microbes^32^, and those pores may be maintained with limited level of oxygen contents and kept with slightly hypoxic conditions.

Generally, the main O_2_-consuming processes in soil are aerobic respiration and nitrification. Soil aerobic respiration is the mineralization of organic C, usually associated with the decomposition of native or added organic matters in soil. Both ammonium (NH_4_^+^) and nitrite (NO_2_^-^ ) oxidation in nitrification would consume O_2_. In this study, the soil NH_4_^+^ content was very low, and nitrification was not the main contributor to the O_2_ consumption. Hence, the anoxia development was more closely linked with the organic matter availability^33^. The addition of C-rich straw enhanced the soil microbial respiration and stimulated a quick consumption of O_2_ in the bulk soil. Additionally, the water absorption by straw could be another reason that affected O_2_ diffusion and caused soil hypoxia^29^. The biochar addition resulted in slightly slower anoxia development than straw treatment, this could be partly due to the biochar adsorption capability of dissolved organic C^34^, which may reduce the availability of organic matter, consequently slower down the O_2_ consumption. Additionally, the porous biochar amendments may have impacts on O_2_ diffusion processes through the influences on the soil pore structure^35^. Both the pores inside biochar and the pores created between biochar particles and soil particles (or the amended straw) may play significant roles in soil pore network, which are very important for gas transport in soils, therefore the biochar addition may enhance the O_2_ diffusions into the soil^36^. Early straw incorporation could allow sufficient time for the mineralization of easily degradable organic matter^37^ under oxic conditions before the BSD process. Additionally, the assisted O_2_ diffusion in the soil by early straw incorporation was indicated by the limited fractions of anoxic area during the early 5 days before BSD (Fig. 3). Furthermore, the early addition of straw may enhance the formation of soil aggregates^38^ that would increase physical protection of organic matter once BSD process began. Therefore, the O_2_ consumption was relatively slower in the treatment that the straw was incorporated earlier before BSD process.

### Linking O_2_ dynamics to GHG emissions

The dynamics of greenhouse gas emissions during the BSD process have been rarely reported. Some studies measured gaseous emission on the top of covered film^17^, which could only indicate the gas fluxes by permeation through the mulch film, and may underestimate the production and emissions of those gases. The present study has, for the first time, monitored the dynamics of direct emissions of CO_2_, N_2_O and CH_4_ during the entire BSD process, and has estimated the significant contribution (2–15%) of CO_2_, N_2_O and CH_4_ fluxes by permeation through the mulch film. The high risk of N_2_O and CH_4_ fluxes were the major concern for the BSD application in vegetable soils.

*N*_*2*_*O emissions* In such high nitrate accumulated soils, denitrification is not limited by the N substrate, and is considered as the major process for N_2_O production in all treatments once the hypoxic fraction prevailed in soil (Fig. 4). Right after BSD process was initiated, straw application enhanced the anoxia development, which, coupled with high nitrate contents in soil, were favorable conditions for the denitrification process^39,40^, mainly due to the unlimited N substrate and extra C supply (electron donors as energy source)^41^. Particularly, the soil O_2_ contents decreased to a certain level and enhanced N_2_O emissions from the straw amended treatments. Soil O_2_ availability in all treatments was identified as the main factor responsible for the initiation of both N_2_O and CH_4_ emissions. Zhu et al.^42^ reported that N_2_O emission accumulated significantly for all soils tested at hypoxic fraction reaching 30% of the soil. In their study, N_2_O emissions were low when hypoxic fraction was higher than 50% or less than 10%. Since those studies were performed using soils amended with animal manure, which had varied degradability of C and N, the dynamics of anoxia development in soils differed with different amendments and soil properties. Interestingly, the peak emissions of N_2_O and peak fractions of hypoxic area were synergized quite well in all treatments at the present study. After the hypoxic area decreased and anoxia developed further, the N_2_O emissions declined, probably due to the further reduction of N_2_O to N_2_ induced by more strict anaerobic conditions.

During the initial period of BSD process, both N_2_O and CO_2_ fluxes increased sharply with the straw incorporation, showing a significant relationship between respiration and denitrification rates^43,44^. The emission peaks of N_2_O from straw amended treatments lasted for 1–3 days, and the peak emission rates were much higher than that of other studies^17^. This difference was partly due to much higher contents of NO_3_^-^ and application rates of straw in the present study. A significant proportion of the straw would be degraded in well-aerated soil within 5 days^29^, thus the earlier incorporation of straw decreased the available C, furthermore, it enhanced the reduction of soil nitrate (Fig. 2), therefore reduce the availability of substrate for denitrification and N_2_O formation as well. Aside from the availability of nitrate and carbon sources, other factors modulating microbial N_2_O production may include soil temperature, soil moisture and pH^45,46^. Soil temperature directly impacts microorganism activity, soil aeration, substrate availability and redistribution. Previous studies reported that N_2_O emissions were synchronized with surface soil temperature^17^. The increases of temperature in this study would possibly boost N_2_O emission rates and cumulative emissions from the soil with relatively high water content^47^.

Biochar’s mitigating effects on N_2_O emissions was still significant under the extreme conditions: high temperature (36 ℃) and high water content (close to saturation) in anaerobic soils. There are several possible explanations. One of the mechanisms may relate to the changes of O_2_ availability in the soil, caused by increased gas diffusivity after amending porous biochar^48,49^. It is also partially due to that biochar reduced both DOC and DON availability for microorganisms (Fig. 2), which depends on the net effects of C sorption by biochar^50,51^ and C release from soil amendments^52^. Finally, pH changes and the toxic effects of compounds formed during pyrolysis could affect microbial activity after biochar addition^19^.

*CH*_*4*_* emissions* Variations of biochemical and microbial parameters can cause different CH_4_ fluxes by influencing CH_4_ production and oxidation processes^53^. One of the key parameters is the soil O_2_ availability^54^. Strictly anaerobic conditions are required for methanogens to produce CH_4_, due to that the key enzymes in methanogenesis are likely inactivated by the presence of O_2_, additionally, methanogens are poor competitors for shared substrates in soils^55^. Therefore, the CH_4_ emissions were not significant until the anoxic fractions of the soil dominated. The hypoxia in agricultural soils usually favored the methanotrophs, which is the major contributor for CH_4_ oxidation^56^, thus CH_4_ emission rates increased dramatically once the hypoxic fraction declined to the minimum.

Wheat straw incorporation significantly stimulated CH_4_ emission, which agrees with previous studies^57,58^. Soil CH_4_ emissions are primarily regulated by the availability of C substrates in soil, and wheat straw as an organic material to soil could provide the available C sources to support the growth of methanogenic populations under anaerobic conditions^59^, especially in the later stage of BSD process with very limited soil O_2_ availability.

The incorporation time of straw had significant impacts on CH_4_ productions and the following emissions as well. As reported by other studies^58,60^, CH_4_ emissions were correlated positively with dissolved organic carbon (DOC), as methanogens are often limited by DOC^61^. In the treatment of WH + 5D, the straw was incorporated 5 days earlier in the soil. Part of DOC contents would be consumed by microbial activities under oxic conditions, resulting in decreased DOC, consequently there were lower CH_4_ emissions from this treatment. The co-application of biochar significantly reduced CH_4_ emissions by 35.4% compared to the treatment of straw addition during the entire BSD process (Fig. 5a). Several studies had reported that the addition of biochar may attenuate the methanogenic activity and improve methanotrophic gene abundance and potential activity^62–64^. Additionally, the oxygen diffusions into the soil may be possibly enhanced by the biochar addition^36^, therefore the biochar amendment could make the soil favorable for methanotrophs but unfavorable for methanogens, consequently led to the declined CH_4_ emissions under BSD conditions.

## Conclusions

The work demonstrated that straw incorporation strategies determined the soil O_2_ dynamics during the BSD treatment. Such amendment-dependent soil O_2_ variations had close linkage with greenhouse gas emissions from vegetable soils. The soil amendment enriched in C-source coupled with the anaerobic conditions created during the BSD treatment may facilitate denitrification and methanogenesis, thereby increasing N_2_O and CH_4_ emissions. Incorporating the straw 5 days before the BSD process could reduce the C availability in soil by oxic degradation and decrease the content of available N by immobilization, therefore, both N_2_O and CH_4_ emissions could be mitigated. The co-application of biochar with straw could reduce the emissions of N_2_O and CH_4_, possibly by extending the hypoxic fractions of soil during BSD process. The results from this study could offer new insights for developing sensitive approaches using soil O_2_ dynamics to predict or mitigate CH_4_ and N_2_O emission during the application of BSD technique.

## Supplementary Information

Supplementary Information

## Data Availability

The datasets used and/or analysed during the current study are available from the corresponding author on reasonable request. All data generated or analysed during this study are included in this published article [and its supplementary information files].
