# Supplementary material for: Implication of O2 dynamics for both N2O and CH4 emissions from soil during biological soil disinfestation
Source: Sci Rep. 2021 Mar 23;11:6590. doi: 10.1038/s41598-021-86026-3 (PMC7988156; doi:10.1038/s41598-021-86026-3)
Supplement: Supplementary file 1 — Supplementary Information [file 41598_2021_86026_MOESM1_ESM.docx]

**Supplementary Information**

Table S1 The basic properties of soil (0–20 cm) and biochar used in the experiment

| **Items** | **Soil** | **Straw** | **Biochar** |
| --- | --- | --- | --- |
| Sand (>0.05 mm), g kg^-1^ | 302.9 | - | - |
| Silt (0.05-0.02 mm), g kg^-1^ | 665.9 | - | - |
| Clay (<0.02 mm), g kg^-1^ | 31.2 | - | - |
| Organic carbon, g kg^-1^ | 33.7 | 43.9 | 52.7 |
| Total nitrogen, g kg^-1^ | 1.7 | 0.8 | 1.1 |
| NH_4_^+^-N, mg kg^-1^ | 1.5 | - | 0.3 |
| NO_3_^-^-N, mg kg^-1^ | 279.6 | - | 0.4 |
| pH | 7.5 | 7.2 | 9.8 |

Table S2 Kinetic parameters and determination coefficient (r^2^) of fitted Arrhenius models for gas permeability of the mulch film

| **Gas** | **E_a_ (J mol^-1^)** | **F_0_ (mg m^-2^ h^-1^ atm^-1^)** | **r^2^** |
| --- | --- | --- | --- |
| CH_4_ | 2.03×10^4^ | 5.55×10^4^ | 0.990 |
| CO_2_ | 2.45×10^4^ | 1.79×10^6^ | 0.986 |
| N_2_O | 2.63×10^4^ | 4.57×10^6^ | 0.980 |

Table S3 Pearson correlations among GHG emission rates and oxygen conditions

| **Correlation Coefficient**  **(P Value)** | **CH_4_** | **CO_2_** | **N_2_O** | **Anoxic**  **fraction** | **Hypoxic**  **fraction** | **Oxic**  **fraction** |
| --- | --- | --- | --- | --- | --- | --- |
| CH_4_ |  | -0.078 | -0.176 | 0.403 | -0.289 | -0.235 |
|  |  | (0.650) | (0.306) | (0.015) | (0.087) | (0.167) |
| CO_2_ |  |  | 0.636 | -0.329 | 0.597 | -0.101 |
|  |  |  | (<0.05) | (<0.05) | (<0.05) | (0.558) |
| N_2_O |  |  |  | -0.176 | 0.601 | -0.279 |
|  |  |  |  | (0.304) | (<0.05) | (0.100) |
